# Comparative Analysis of Bone Structural Parameters Reveals Subchondral Cortical Plate Resorption and Increased Trabecular Bone Remodeling in Human Facet Joint Osteoarthritis

**DOI:** 10.3390/ijms19030845

**Published:** 2018-03-14

**Authors:** Cordula Netzer, Pascal Distel, Uwe Wolfram, Hans Deyhle, Gregory F. Jost, Stefan Schären, Jeroen Geurts

**Affiliations:** 1 Department of Spine Surgery, University Hospital of Basel, 4031 Basel, Switzerland; Cordula.Netzer@usb.ch (C.N.); Pascal.Distel@unibas.ch (P.D.); Gregory.Jost@usb.ch (G.F.J.); Stefan.Schaeren@usb.ch (S.S.); 2 Department of Biomedical Engineering, University Hospital of Basel, 4123 Allschwil, Switzerland; hans.deyhle@unibas.ch; 3 School of Engineering & Physical Sciences, Mechanical, Process & Energy Engineering, Heriot-Watt University, Edinburgh EH14 4AS, UK; u.wolfram@hw.ac.uk

**Keywords:** osteoarthritis, lumbar spine, facet joint, subchondral bone, computed tomography

## Abstract

Facet joint osteoarthritis is a prominent feature of degenerative spine disorders, highly prevalent in ageing populations, and considered a major cause for chronic lower back pain. Since there is no targeted pharmacological therapy, clinical management of disease includes analgesic or surgical treatment. The specific cellular, molecular, and structural changes underpinning facet joint osteoarthritis remain largely elusive. The aim of this study was to determine osteoarthritis-related structural alterations in cortical and trabecular subchondral bone compartments. To this end, we conducted comparative micro computed tomography analysis in healthy (*n* = 15) and osteoarthritic (*n* = 22) lumbar facet joints. In osteoarthritic joints, subchondral cortical plate thickness and porosity were significantly reduced. The trabecular compartment displayed a 42 percent increase in bone volume fraction due to an increase in trabecular number, but not trabecular thickness. Bone structural alterations were associated with radiological osteoarthritis severity, mildly age-dependent but not gender-dependent. There was a lack of association between structural parameters of cortical and trabecular compartments in healthy and osteoarthritic specimens. The specific structural alterations suggest elevated subchondral bone resorption and turnover as a potential treatment target in facet joint osteoarthritis.

## 1. Introduction

Facet joint osteoarthritis (FJOA) is a prominent radiological feature of several degenerative spine disorders including spinal stenosis, spondylolisthesis, and intervertebral disc degeneration [1,2,3,4]. Lumbar FJOA is highly prevalent, but not necessarily symptomatic, in Western and Asian communities, occurring in over 50 percent of the population over the age of 50 [5,6]. Especially in the older individuals, the presence and extent of lumbar FJOA is strongly associated with low back pain [7]. Management of facet-mediated pain commonly involves intra-articular injection of analgesic agents and spinal fusion as conservative and surgical treatment approaches, respectively [8,9]. Pharmacological treatment strategies for FJOA are lacking, owing to the limited understanding of the pathomechanisms and structural tissue changes underpinning FJOA.

Owing to the reported associations of FJOA and low back pain, the majority of histopathological characterizations of facet joints and their capsular tissues have focused on the identification of pain-sensing nerve structures or pain mediators [10,11,12,13]. Both nociceptive nerve fibers and neuromodulators, such as substance P and nerve growth factor, have been identified in capsular tissues of degenerative facet joints. It has been shown that enhanced remodeling of subchondral bone and marrow tissues is a predominant feature in the trabecular compartment in FJOA [14,15,16]. Elevated active bone-forming osteoblasts accompanied by excessive collagen fiber deposition were indicative of a shift towards bone formation [14]. Foci of new bone formation were described to co-localize with granulation tissue replacing fatty marrow in FJOA and ankylosing spondylitis [15]. The specific changes in bone structural parameters due to enhanced remodeling have not been identified thus far.

An in-depth characterization of bone structural parameters of cervical and lumbar facet joints from healthy subjects using micro-computed tomography (μCT) revealed age- and gender-related differences in the subchondral cortical plate (SCP) and subchondral trabecular bone (STB) [17]. Porosity of the SCP was higher in females than males, but did not vary during aging. Aging-related trabecular bone loss was evident in both genders and a steeper decline occurred in females. Trabecular bone loss was observed prior to cartilage degeneration in early experimental FJOA, but SCP and STB structural changes were not determined in advanced disease [18].

In the present study, we aimed to (1) perform an in-depth μCT study to determine structural changes of SCP and STB in human lumbar FJOA compared with healthy subjects; and (2) evaluate whether structural changes are associated with osteoarthritis severity, age, and gender.

## 2. Results

### 2.1. Facet Joint Osteoarthritis Is Characterized by Higher Trabecular Bone Volume and Less Subchondral Cortical Plate Thickness

**Figure 1:**
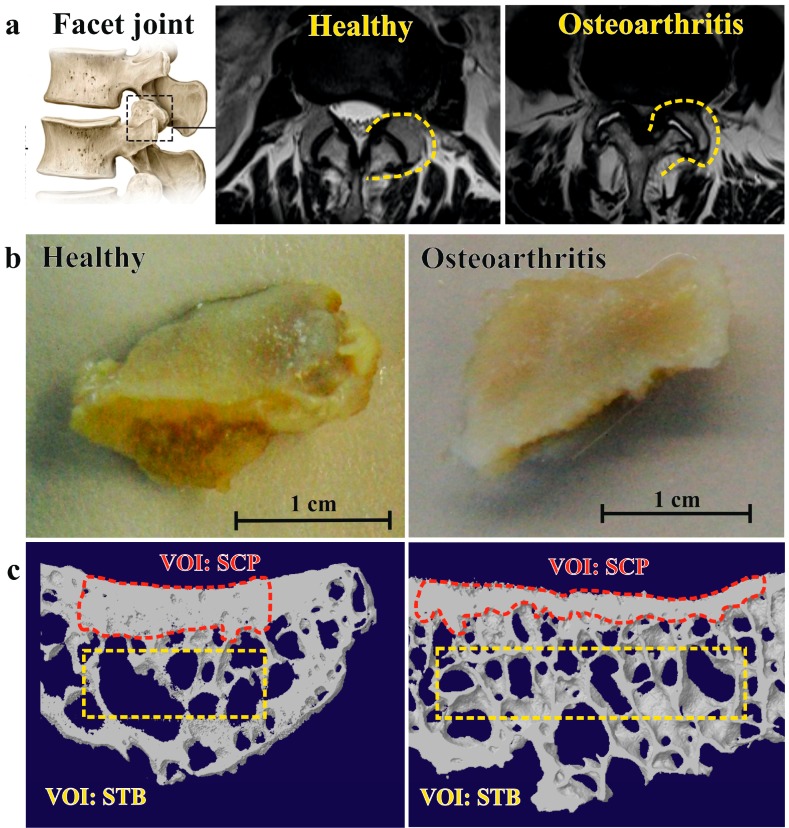
Schematic overview of the study. (**a**) Facet joint anatomy (left) and grading of facet joint osteoarthritis severity on axial MRI images. Healthy joints (Weishaupt grade 0) display no radiological abnormalities or joint space narrowing. Facet joints with severe osteoarthritis (Weishaupt grade 3) display joint effusion (white signal in joint space), joint hypertrophy, and osteophyte formation; (**b**) top view of clinical specimens from healthy (Weishaupt grade 0) and osteoarthritic (Weishaupt grade 3) facet joints obtained by partial facetectomy; (**c**) representative three-dimensional reconstruction images of CT scans indicating selection of volumes of interest (VOI) for analysis of subchondral cortical plate (SCP) and subchondral trabecular bone (STB) compartments.

Twenty-two patients with lumbar spinal stenosis undergoing fusion surgery with partial facetectomy were recruited into the study. Magnetic resonance imaging (MRI)-based grading (Figure 1a) showed that the majority of patients displayed severe (Weishaupt grade 3, *n =* 12) or moderate (Weishaupt grade 2, *n =* 5) osteoarthritis at the affected lumbar spine level (L3–L5). Mild (Weishaupt grade 1) or absent radiological osteoarthritis was detected in three and two subjects, respectively. Structural parameters of subchondral trabecular and cortical bone compartment were determined by μCT scanning of clinical facet joint specimens (Figure 1b) followed by manual image segmentation and analyses (Figure 1c). Bone structural parameters of osteoarthritic specimens were compared with pooled data (L4/L5) from healthy controls with comparable age range (43–96) and gender distribution (Table 1).

**Figure 2:**
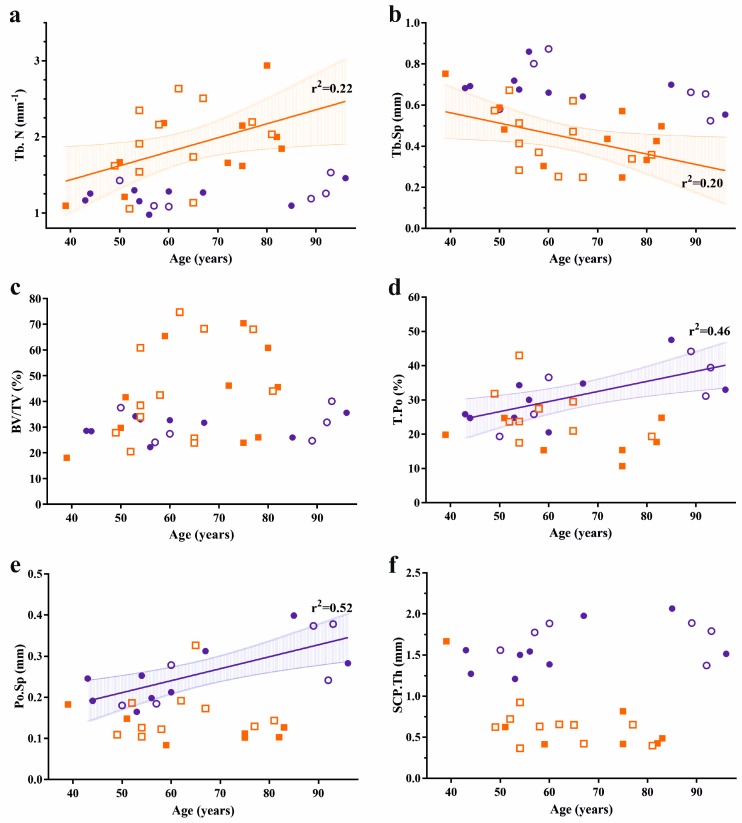
Age- and gender-related distribution of bone structural parameters in lumbar facet joints. Healthy facet joints are marked blue, FJOA specimens are marked orange. Males are represented by filled symbols, females by open symbols. Significant correlations with age are marked by regression lines with 95% confidence intervals and *r*^2^-values for healthy (blue) and osteoarthritic (orange) specimens. Distributions are shown for (**a**) trabecular number; (**b**) trabecular separation; (**c**) bone volume fraction; (**d**) cortical plate porosity; (**e**) cortical pore space; and (**f**) cortical plate thickness.

Subchondral trabecular bone volume fraction of FJOA specimens was 1.4-fold higher compared with healthy controls. Increased bone volume fraction corresponded with a significant 1.5-fold increase of trabecular number, while trabecular thickness remained unchanged. Due to the increase in trabecular number, trabecular spacing was significantly less in FJOA. Significant differences in trabecular pattern factor between both groups pointed towards higher intertrabecular connectivity in FJOA. In contrast, subchondral cortical plate thickness was reduced 2.5-fold in osteoarthritic specimens. Total porosity and pore space size were both significantly less in FJOA (Table 1). Age- and gender-related distribution of the data is plotted in Figure 2.

### 2.2. Bone Structural Parameters Associate with Osteoarthritis Severity and Age, but Are Not Gender-Dependent

Next, we conducted correlation analyses to evaluate whether bone structural changes in FJOA depend on osteoarthritis severity, age, and gender. Pearson correlation analysis revealed significant associations between Weishaupt grade and structural parameters in the STB, but not SCP compartment (Table 2). Osteoarthritis severity showed a moderate correlation with age (*r* = 0.47), but was gender-independent.

Linear regression analysis was conducted to assess whether bone structural parameters from osteoarthritic and healthy facet joints were age-dependent (Figure 2). Age accounted for 22 and 20 percent of the variation in trabecular number (*r*^2^ *=* 0.22, *p* = 0.03) and trabecular separation (*r*^2^ *=* 0.22, *p* = 0.03) in FJOA specimens, respectively. SCP bone parameters were not age-dependent in osteoarthritic specimens. STB parameters were not correlated with age in healthy facet joints. In contrast, age accounted for 46 and 52 percent of the variation in porosity (*r*^2^ *=* 0.46, *p* = 0.006) and pore space (*r*^2^ *=* 0.52, *p* = 0.003), respectively. None of the bone structural parameters were associated with gender in both groups.

### 2.3. Lack of Association between Trabecular and Cortical Structural Parameters in Facet Joints

Finally, we sought to investigate whether there was an association between subchondral cortical plate thickness and STB and SCP parameters in healthy and osteoarthritic facet joints (Table 3). Subchondral plate thickness was not correlated with any parameter in the STB compartment. Subchondral plate porosity and pore space were significantly correlated with subchondral plate thickness in healthy joints only.

## 3. Discussion

In the present study, we performed a comparative μCT analysis of subchondral cortical and trabecular bone parameters of lumbar facet joints in healthy subjects and spine osteoarthritis patients. Corroborating findings from previous two-dimensional histopathological studies [14,16] in a three-dimensional analysis, we found FJOA was characterized by an increase of subchondral trabecular bone volume due to a higher trabecular number, but not thickness. In contrast, the subchondral cortical plate was significantly thinner in osteoarthritic compared to healthy facet joints. Surprisingly, subchondral cortical and trabecular bone parameters did not reveal a strong intra-individual correlation in healthy or osteoarthritic joints. The specific structural alterations in FJOA were dependent on osteoarthritis severity and age, but not gender.

In an independent study, Duan and co-workers used clinical resolution MRI to determine age- and gender-related variation of SCP thickness in facet joints of healthy volunteers under the age of 60 [19]. The average thickness reported was 1.56 mm (range L3–L5, 1.21–2.12 mm), which is well in range with our μCT-based assessment (1.62 ± 0.26 mm). As our results demonstrate that a strong decrease of SCP thickness occurs, irrespective of osteoarthritis severity, it would be interesting to see whether this prominent radiological feature of human FJOA could detected by routine MRI imaging. Elevated resorption and thinning of the SCP is commonly observed at an early stage in experimental models of knee osteoarthritis, followed thickening and sclerosis in end-stage disease [20,21,22]. Similar findings have been described in experimental models of facet joint degeneration and osteoarthritis. Facet joint degeneration induced by intra-articular injection of mono-iodoacetic acid in rats resulted in 20–30% reduction of cortical thickness one week post-injection, followed by progressive cartilage loss up to three weeks thereafter [18]. Similarly, ovariectomized mice displaying enhanced loss and collapse of cortical bone in facet joints, rapidly developed severe cartilage degeneration. Interestingly, the development of an osteoarthritic phenotype could be rescued by estrogen treatment that prevented progressive cortical bone loss [23]. While cross-sectional and longitudinal clinical imaging data of FJOA are crucially lacking, our results suggest that loss of cortical plate thickness is a prominent feature in human disease as well. Elevated osteoclast numbers at the osteochondral junction in FJOA and ankylosing spondylitis have been described previously [16], providing histological support for the involvement of cortical plate loss in human facet joints. Interestingly, SCP thickness did not vary significantly with osteoarthritis grade (Table 2), which suggests that loss of cortical plate thickness might be a common feature of early FJOA.

Remodeling of the STB compartment in FJOA was characterized by larger trabecular number, while trabecular thickness remained unchanged. These structural alterations can be well explained from biomechanical and biological perspectives. Mathematical simulations of trabecular bone tissue have shown that bone strength and stiffness is enhanced through higher trabecular number [24], which would be required to adapt to increased loading conditions in degenerative facet joints. Formation of new bone tissue can be achieved through either static or dynamic osteogenesis [25,26]. Dynamic osteogenesis involves osteocyte-induced activation of pre-existing bone-lining osteoblasts leading to thickening of trabeculae. Static osteogenesis instead gives rise to formation of new trabeculae of less quality through recruitment of osteoprogenitors by endothelial-derived growth factors. Previous histological studies have demonstrated extensive de novo woven bone formation by granulation tissue in subchondral marrow spaces in human FJOA specimens [14,15]. Structural and histological data therefore suggest that static osteogenesis underpins specific bone remodeling in FJOA. Moreover, the involvement of static, rather than dynamic, osteogenesis could indicate that the function or viability of osteocytes is impaired in FJOA. A histological analysis of osteocyte lacunae in healthy and osteoarthritic specimens could provide further support for the role of static osteogenesis in the pathogenesis of FJOA.

Increased cortical plate resorption and turnover and remodeling of the STB compartment as prominent features raise the question of whether or not the presence of FJOA might be detected through assessment of biochemical markers of bone remodeling. A cross-sectional study evaluating serum and urine biomarkers in patients with FJOA or intervertebral disc degeneration did not report a significant increase in type I collagen between groups [27]. Instead, a biomarker of inflammation (hyaluronan) was found predictive for the presence of FJOA. These findings were however confounded by the presence of knee, hip, or hand OA. It would therefore be interesting to determine biochemical markers of bone remodeling in isolated spine pathologies and healthy controls.

The involvement of elevated cortical plate resorption and trabecular bone turnover and remodeling suggest that targeting bone tissue might be a treatment strategy in FJOA. There is increasing evidence and consensus that three different clinical OA subpopulations exist displaying either traumatic, inflammation-driven or bone-driven pathophysiology [28]. Numerous pre-clinical and clinical studies have shown that the bone-driven phenotype in knee OA can be treated using a variety of bone-acting drugs including anti-resorptive agents and bone anabolic agents (reviewed in [29]). While it has been shown that long-term anti-resorptive treatment with alendronate in osteoporosis patients slowed the progression of vertebral osteophytes and intervertebral disc narrowing [30], effects on facet joints remained unexplored. Together, previous histological findings and results from this study suggest that experimental studies evaluating bone-targeting agents in facet joint osteoarthritis are therefore warranted.

We acknowledge a number of limitations to this study. The results of this cross-sectional study were not adjusted for potential confounders such as facet joint tropism, orientation, and spine level. While these factors would not influence the comparative analysis of healthy and osteoarthritic facet joints, these factors are known to determine the prevalence of FJOA [31,32]. Age- and gender-dependent associations in healthy and degenerated conditions have to therefore be carefully interpreted. Adjustment for potential confounders is further limited by the sample size of this study. Lastly, differences in sample harvesting and processing between partial facetectomy specimens from spinal stenosis patients and cylindrical drill cores from healthy cadaveric controls prohibited precise matching for age, gender, spine level, and volume of interest in this study. We therefore used a global approach, where measurements in healthy controls [17] were averages of pooled data from multiple spine levels (L3–L5) for each patient.

In conclusion, we have identified thinning of the subchondral cortical plate and increase of trabecular number as specific bone structural alterations in osteoarthritis compared with healthy facet joint specimens. This study provides morphological evidence for a bone-driven pathophysiology in facet joint osteoarthritis and with this a plausible rationale for a treatment strategy targeting elevated bone resorption and turnover.

## 4. Materials and Methods

### 4.1. Collection of Clinical Specimens and Grading of Facet Joint Osteoarthritis

Osteoarthritic lumbar facet joints (L3–L5) were obtained by facetectomy from 22 patients undergoing transforaminal lumbar interbody fusion surgery for lumbar spinal stenosis. Twelve patients were female and the average age was 63.6 years (range 39–82). None of the patients received treatment with bone-acting anti-osteoporotic drugs. All patients underwent routine preoperative X-ray standing in upright position and MRI of the lumbar spine. Facet joint osteoarthritis was graded retrospectively on MRI using the Weishaupt grading system (0 = none, 1 = mild, 2 = moderate, 3 = severe osteoarthritis) [33]. Joint space narrowing, osteophyte formation, and hypertrophy of articular processes—as well as subchondral erosions and cysts—were evaluated on standard T1-weighted and T2-weighted sequences in sagittal and axial orientation.

Clinical specimens were immediately fixed in formalin for two days and thereafter stored in 70% ethanol at 4 °C. Written confirmed consent was obtained from all patients and this study has been reviewed and approved by the local ethical committee (no. 147/12, 29 May 2012).

### 4.2. Micro Computed Tomography Scanning

Fixed specimens were immersed in 70% ethanol and μCT scans were performed at a resolution of 20 μm/pixel using a Skyscan 1174 (Bruker Corporation, Kontich, Belgium). Scanning settings were: 50 kV, 800 mA, 3000 ms exposure time, 360° scan, 0.5 mm aluminium filter, 0.3° rotation angle, averaging three frames. Three-dimensional reconstructions were performed using NRecon version 1.6.6 (Bruker Corporation).

### 4.3. Micro Computed Tomography Analysis

Bone structural parameters were determined using CT-Analyser version 1.13 (Bruker Corporation). For analysis of the STB compartment, volumes of interest (VOI) were placed in the central region of the specimen at least 0.5 mm under the bottom portion of the SCP (Figure 1c, yellow dashed rectangle). VOIs were placed well away from the edges of the specimen and cortical bone. The following parameters were evaluated: bone volume fraction (BV/TV), trabecular thickness (Tb.Th), trabecular number (Tb.N), trabecular spacing (Tb.Sp), trabecular pattern factor (Tb.Pf), and degree of anisotropy (DA).

For analysis of SCP compartment, the cortical was segmented by manual tracing (Figure 1c, red dashed shape) and total porosity (T.Po) and pore space (Po.S) were determined. Binary images were further processed using ImageJ (Version 1.49v, National Institute of Health, Bethesda, MD, USA) to close pores prior to measurement of the SCP thickness (SCP.Th). The accuracy (coefficient of variation <0.5%) and reproducibility (intraclass correlation coefficient: 0.999–1.00) of volumetric assessments are very high for routine desktop μCT scanners [34].

### 4.4. Comparative Analysis of Bone Structural Parameters of FJOA and Cadaveric Controls

Bone structural parameters from SCP and STB VOIs from FJOA were compared with a subset L4/L5 facet joints from 15 cadaveric controls with comparable age range and gender distribution [17]. Nine controls were female and the average age was 66.6 years (range 43–96). Specimens were visually inspected to exclude joints displaying hypertrophy and osteophytes. Cylindrical cores from the central portion of L4/L5 facet joints were scanned at a resolution of 30 μm/pixel. STB VOIs were placed well away from cortical bone and SCP VOIs were traced manually. Measurements of bone structural parameters were averaged for L4/L5 levels and compared with VOIs from FJOA specimens.

### 4.5. Statistical Analysis

Statistical analyses were performed using GraphPad Prism (v7.00, GraphPad Software Inc., La Jolla, CA, USA). Variables followed a normal distribution as assessed by D’Agostino and Pearson omnibus normality test. Data are reported as mean ± standard deviation. Significant differences between healthy controls and FJOA were calculated using Student’s *t*-test. Association between bone structural parameters and MRI-based osteoarthritis grade was determined using Spearman correlation analysis. Association between subchondral thickness and bone structural parameters was determined by Pearson correlation analysis. Correlation coefficients are given as Spearman or Pearson *r* with 95% confidence intervals. The correlation between age and bone structural parameters was determined by linear regression. We used a significance threshold of *p =* 0.05

## Figures and Tables

**Table 1:** Subchondral bone structural parameters in healthy and osteoarthritic lumbar facet joints.

Parameter	Healthy	Osteoarthritis	*p*-Value
Age in years	66.6 ± 18.9	63.6 ± 12.5	0.563
Gender (f/m)	9/6	12/10	0.398
**Subchondral Trabecular Bone**			
Trabecular number in mm^−1^	1.24 ± 0.15	1.87 ± 0.50	<0.0001
Trabecular separation in mm	0.685 ± 0.099	0.443 ± 0.145	<0.0001
Trabecular thickness in mm	0.240 ± 0.018	0.232 ± 0.064	0.637
Bone volume fraction in %	30.5 ± 5.2	43.5 ± 18.4	0.012
Trabecular pattern factor in mm^−1^	−0.080 ± 0.98	−4.54 ± 7.04	0.021
Degree of anisotropy	9.6 ± 15.6	6.5 ± 8.5	0.453
**Subchondral Cortical Plate**			
Total porosity in %	31.5 ± 8.2	22.9 ± 7.8	0.0054
Pore space in mm	0.26 ± 0.08	0.15 ± 0.06	<0.0001
Cortical thickness in mm	1.62 ± 0.26	0.62 ± 0.31	<0.0001

**Table 2:** Correlation between Weishaupt grade and bone structural parameters in FJOA.

Parameter	Spearman *r* [95% CI]	*p*-Value
Age	0.47 [0.04, 0.75]	0.028
**Subchondral Trabecular Bone**		
Trabecular number	0.55 [0.15, 0.79]	0.009
Trabecular separation	−0.57 [−0.80, −0.18]	0.006
Trabecular thickness	0.27 [−0.18, 063]	0.219
Bone volume fraction	0.52 [0.11, 0.78]	0.014
Trabecular pattern factor	−0.42 [−0.72, 0.02]	0.053
Degree of anisotropy	0.37 [−0.07, 0.69]	0.260
**Subchondral Cortical Plate**		
Total porosity	−0.07 [−0.55, 0.45]	0.798
Pore space	−0.48 [−0.79, 0.04]	0.063
Cortical thickness	−0.32 [−0.71, 0.22]	0.221

**Table 3:** Correlation between subchondral cortical plate thickness and bone structural parameters.

	Healthy		Osteoarthritis	
Parameter	Pearson *r* [95% CI]	*p*-Value	Pearson *r* [95% CI]	*p*-Value
**Subchondral Cortical Plate**				
Total porosity	0.73 [0.34, 0.90]	0.002	−0.07 [−0.55, 0.44]	0.797
Pore space	0.75 [0.39, 0.91]	0.001	0.28 [−0.25, 0.68]	0.291
**Subchondral Trabecular Bone**				
Trabecular thickness	−0.17 [−0.63, 0.37]	0.543	−0.04 [−0.53, 0.46]	0.875
Trabecular number	−0.22 [−0.66, 0.33]	0.427	−0.48 [−0.79, 0.03]	0.068
Trabecular separation	0.08 [−0.45, 0.57]	0.772	0.48 [−0.02, 0.79]	0.062
Bone volume fraction	−0.29 [−0.69, 0.26]	0.299	−0.25 [−0.66, 0.28]	0.347

## Abbreviations

FJOA	Facet joint osteoarthritis
μCT	Micro computed tomography
SCP	Subchondral cortical plate
STB	Subchondral trabecular bone
